# Injuries of the Eye

**Published:** 1882-02

**Authors:** C. A. Lambert

**Affiliations:** Surgeon in Charge, Goshen, Ind., Eye and Ear Infirmary


### ARTICLE V.

INJURIES OF THE EYE. Clinical History of some recent Cases in the Practice of C. A. LAMBERT, M.D., Surgeon in Charge, Goshen, Ind., Eye and Ear Infirmary. Reported before Elkhart County Medical Association, December Meeting, 1881.

*Case* I.—*Traumatic Aniridia.*—Mr. J. W. J., æt. twenty-six; occupation, teamster; reported at office October 19, 1881.

*History.*—October 11, eight days ago, while at work skidding logs, was accidentally struck a violent blow by a skid, fracturing malar bone at outer canthus, and cutting the cuticle so as to produce considerable hæmorrhage. The eye must have received at the same time much of the force of the blow. He was knocked to the ground and stunned, and came near vomiting after recovering from the concussion.

*Status Præsens.*—Wound over malar bone healed. Can feel injured bone, and trace fracture of malar bone well into orbital plates, above and below. Complains of ciliary, temporal, and general hemicranial neuralgia, loss of appetite, restlessness; the neuralgic pain is very acute. Eyeball, T+2. Sclera greatly injected. No rupture of cornea nor sclerotic. S=O. Cornea clear. Iris completely gone; lens dislocated upward and backward well into vitreous, and completely opaque; cannot detect any rupture of capsule. The lens inclines backward at an angle of about 30°; aqueous very cloudy, and contains strips of coagulum. By oblique illumination can, inferiorly, discern a dark red ring, but cannot get any red reflex. There are general symptoms of choroiditis.

*Treatment.*—Eserine sol., and dry compress for ten days, when patient was discharged, with eye in the following condition. Light enters the eye readily at lower margin of lens, and with ophthalmoscope can get a brilliant red reflex. Can count fingers at five feet, when held vertically and well above level of the eye. I expected to have to remove lens, but the eye has remained quiescent up to this time, three months after the injury; and in case the patient returns, I respectfully invite some suggestions from the readers of this report, and they will be thankfully received. I am satisfied the iris did not escape from the eye, for there was no rupture of cornea or sclerotic, and I suppose the iris is in the fundus, and will prove a source of future trouble. Then, again, as the lens has nothing supporting its present position but the vitreous, it cannot fail to act as a future source of mischief to the eye. The fine question arises, of the propriety of its removal when the iris is completely gone.

*Case* II.—*Hæmorrhage into Anterior Chamber from Traumatism.*—Mr. Jacob Groff, æt. forty-three.

*History.*—Received injury to left eye two days ago, while hauling logs. He struck out at an unruly ox, and the whip-lash came home to him, striking the left eye; says that the blow on the eye made him sick, and that he came near vomiting, and that the eye became blind at once.

*Status Præsens.*—Temporal and ciliary neuralgia. Lachrymation, S=O. Anterior chamber filled with a blood coagulum. Cannot see any of the iris.

*Treatment.*—Compress and atropia for ten days. Eye thoroughly recovered. $S=\frac{20}{20}$.

*Case* III.—Peter Surfus, æt. fifty-seven; occupation, farmer.

*History.*—Four days ago, while feeding flax-straw into the cylinder of a threshing machine, and just as he was stepping aside to give place to a relief, a piece of split flax-straw, about one-fourth inch long, flew from the cylinder with such tremendous force as to penetrate the cornea.

*Status Præsens.*—Circumcorneal injection, some photophobia and lachrymation, and some slight ciliary pain; could see the piece of straw resting on anterior surface of iris, and directly across the pupil, in a horizontal position.

*Operation.*—Patient anæsthetized with chloroform; applied fixation forceps; rotated eye a little inward and upward, and then introduced a straight iridectomy knife to its full width into outer and lower quadrant of corneo-scleral formation; allowed the aqueous to escape freely, anticipating that the straw would follow at least a few lines toward the incision; but the contrary happened—the straw receded. I then introduced a pair of iridectomy forceps, with the beaks tightly closed, and by a very patient wriggling movement, and carefully pressing down the iris, the forceps at last reached the straw. I felt that I had but this one chance, and congratulated myself that I was able to seize the straw and withdraw it on the first effort. We took the precaution to instil sol. atropia before making the incision. The usual dressing, as in iridectomy operation, was applied, and the eye made a complete and rapid recovery. $S=\frac{20}{20}$.

*Case* IV.—*Traumatic Lens Extraction and Iridectomy.*—James Mc., æt. twenty-one years; occupation, farmer.

*History.*—Three weeks ago, while currying a horse, received injury to one of his eyes. The horse suddenly raised his limb, and the knee struck the young man's eye a violent blow.

*Status Præsens.*—Three weeks after injury, has a partially absorbed blood coagulum in anterior chamber, which extends from the wound up to and into the pupil, hiding corresponding portion of the iris; wound partially healed. T–3. $S=\frac{1}{20}$. Pupil dilated upward under use of atropia; attached somewhat below. I think the lens has escaped from the ball; can get no reflex from the fundus. The blow ruptured the cornea around margin, from base to vertical meridian around to outer extremity of horizontal, and extending into sclerotic in same course for 3'''.

*Treatment.*—Tonics; ferri et ammoniæ citratis in port wine, collyr. atropiæ sul., shade for the eye, and cautioned him of any excesses. April 22, wound nearly healed; hæmorrhage being rapidly absorbed; iris has an appearance as though an iridectomy had been made at lower border at the time of the injury. Lens certainly has escaped; vision improves; can see choroidal discoloration in scleral wound. Patient can, with this eye (the right one) count fingers and see quite small objects. The lens escaped through wound in cornea and sclerotic. There is no retinal trouble; the ball is not sensitive, but is somewhat atrophied; but remains quite natural in appearance, and sight constantly improves.

*Case* V.—*Paralysis and Loss of Sight from Injury.*—Master Chas. H., æt. fourteen years; healthy.

*History.*—Had received injury five weeks ago to right eye; was struck accidentally with a stick on outer and upper portion of globe; accident was followed by violent spasms, and frequent vomiting for some twenty-four hours after injury.

*Status Præsens.*—Exophthalmos; iris widely dilated; lids discolored, and complete ptosis. S=O. Has ciliary and temporal neuralgia. Vitreous hazy; chronic retinitis; has paralysis in second, third, fourth, sixth, and ophthalmic branch of fifth, with its branches, lachrymal, frontal and nasal; the branches of nasal, ganglionic, ciliary and infra-trochlear. Lids and eye-ball completely immovable.

*Treatment.*—Comp. syr. phos. ferri quiniæ et strych.; collyr., atropiæ sul.; but the chief reliance was on a current of electricity used twice each week. Condition March 25, has regained all motions of lids and eye-ball excepting upward; no other deformity. Pupil dilated. Has white atrophy of optic papillæ. S=O.

*Case* VI.—*Paralysis of all the Muscles of Lids and Eye-ball.*—John Mann, æt. nineteen years; farmer; healthy.

*History.*—Two weeks ago, when at home, and while groping around early in the morning, in search of his boots, he accidentally, when stooping, jammed the lower quadrant of the left eye against the spool-holder on the sewing-machine. The blow made him violently sick for about twenty-four hours.

*Status Præsens.*—Complete paralysis of all the muscles of motion of eye-ball and lids; marked ptosis and exophthalmos. $S=\frac{20}{20}$.

*Treatment.*—Comp. syr. phos. iron, quiniæ et strych., and a current of electricity twice each week. After three weeks' treatment, the effects of the injury passed entirely away, and the recovery was perfect.

---

GOSSYPIUM HERBACEUM.—The following are the results of a series of experiments upon the physiological action of this drug, by J. Charles Martin, M.D. With frogs a large amount of the drug was necessary to produce definite results, 20 to 30 M. of the fluid extract being required. Stupor, inattention to surrounding objects, lowered perception of impression, and diminished muscular activity were the prominent symptoms developed. Nerves such as the sciatic preserve their irritability, as do the muscles also. Sensibility is only gradually lost as larger amounts are given. Reflex action is not destroyed, and there is no change in cardiac function, neither depression nor stimulation. When similar injections were made in frogs, the medullæ of which were divided, and the sciatic nerve on one side of each isolated by ligating all the tissues, reflex functions, motility and sensibility were found to be unimpaired, and were exactly the same in each limb. Therefore it is concluded that the stupor noted in the former cases was due to the cerebral effect of the drug.

Experiments upon pregnant rabbits, which usually conceive and abort with equal facility, showed no effect from the medicine upon the uterus. Even toxic doses produced no hyperæmia or other effect on this organ. The symptoms manifested were gradually increasing stupor, and in the same ratio impairment of motility and sensibility.—*The Am. Jour. Medical Sciences, January.*

